# Unraveling the Dyslipidemic Landscape in Moyamoya Disease: OxLDL as a Key Biomarker

**DOI:** 10.1111/cns.70441

**Published:** 2025-05-14

**Authors:** Chaofan Zeng, Haoyuan Chen, Jie Liu, Youyuan Bao, Xudong Sun, Fanbo Meng, Yimeng Xue, Yunhao Cui, Qianjun Zhao, Jing Zhang, Hao Li, Dong Zhang, Rong Wang, Yan Zhang, Guojun Zhang, Jizong Zhao, Qian Zhang

**Affiliations:** ^1^ Department of Neurosurgery, Beijing Tiantan Hospital Capital Medical University Beijing China; ^2^ China National Clinical Research Center for Neurological Diseases Beijing China; ^3^ Center of Stroke Beijing Institute for Brain Disorders Beijing China; ^4^ Beijing Key Laboratory of Translational Medicine for Cerebrovascular Disease Beijing China; ^5^ Department of Clinical Diagnosis, Laboratory of Beijing Tiantan Hospital Capital Medical University Beijing China; ^6^ Department of Neuropathology Beijing Neurosurgical Institute Beijing China; ^7^ Capital Medical University Beijing China; ^8^ Department of Neurosurgery Beijing Hospital, National Center of Gerontology Beijing China

**Keywords:** biomarker, dyslipidemia, moyamoya disease, oxLDL

## Abstract

**Aims:**

The pathogenic mechanisms of moyamoya disease (MMD) remain unrecognized. Although genetic predisposition and hemodynamic changes have been focused on, emerging evidence suggests dyslipidemia may also contribute to MMD. Here, we performed a comprehensive analysis of lipid profiles, aiming to elucidate potential mechanisms in MMD.

**Methods:**

In this prospective case–control study, 222 MMD patients and 231 healthy controls (HCs) were enrolled. The comprehensive lipid profiling was performed, encompassing standard lipids, apolipoproteins, oxidized low‐density lipoprotein (oxLDL), and small dense LDL (sdLDL). Statistical models of weighted quantile sum (WQS) and Bayesian kernel machine regression (BKMR) were applied to capture individual and joint lipid effects on MMD risk.

**Results:**

Compared with HCs, MMD patients exhibited significantly higher oxLDL, sdLDL, and lipoprotein(a) (*p* < 0.05). OxLDL emerged as a robust independent risk factor for MMD (adjusted OR 1.146, 95% CI 1.102–1.210, *p* < 0.001). WQS analysis further identified oxLDL as the single greatest contributor to MMD risk, with additional support from BKMR showing marked synergistic interactions between oxLDL and homocysteine.

**Conclusions:**

The study revealed a comprehensive dyslipidemic landscape in MMD, highlighting oxLDL as a pivotal biomarker. The results underscored the significance of lipid metabolism in MMD pathogenesis, warranting further investigation to guide novel diagnostic and therapeutic strategies.

## Graphical abstract

Our study systematically characterized the dyslipidemic profiles of patients with moyamoya disease (MMD) and uncovered a pivotal role of oxLDL in MMD pathogenesis. Higher level of oxLDL was closely associated with increased disease risk, indicating the potential value as diagnostic and therapeutic targets. These findings highlighted the significance of lipid metabolism in guiding future risk stratification and management strategies in MMD.

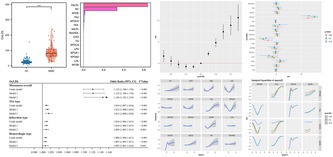

## 1 Introduction

Moyamoya disease (MMD) is a cerebrovascular disorder characterized by progressive stenosis of the intracranial internal carotid arteries and the formation of fragile collateral vessels, leading to ischemic or hemorrhagic strokes [1]. Although the precise mechanisms of MMD remain elusive, multiple lines of evidence point to a complex interplay among genetic susceptibilities, aberrant angiogenesis, and immune‐inflammatory processes that drive the disease course [2, 3]. However, comprehensive studies investigating the lipid profiles in MMD and its potential contribution to disease risk are limited.

Emerging evidence suggested that dyslipidemia and oxidative stress may play critical roles in the vascular remodeling and inflammatory processes associated with MMD [4, 5, 6]. Recent findings have documented elevated total cholesterol (CHO), triglycerides (TG), and lipoprotein(a) (LPa) in MMD [7, 8]. Advanced cerebrovascular lipidomics has identified novel biomarkers linked to inflammation, endothelial dysfunction, and vascular remodeling, suggesting that lipid abnormalities in MMD extend beyond the routine circulating lipids [6]. Nevertheless, the traditional lipid indicators have not fully explained the striking vascular changes observed in MMD. Emerging data have shown that oxidized LDL (oxLDL), small dense LDL (sdLDL), and other lipid fractions might be implicated in endothelial dysfunction and pathological neovascularization [9, 10]. The role of genetic polymorphisms, such as *RNF213* and *APOE*, further supports the notion that dyslipidemia in MMD involves both genetic and acquired mechanisms [11, 12]. Understanding this complex metabolic interplay could have significant implications for precision management and therapeutic development.

In the present study, we conducted a comprehensive case–control study to explore the full spectrum of lipid disturbances in MMD and to elucidate their relationships with clinical subtypes, genetic polymorphisms, and mechanistic interactions. Employing advanced analytical approaches, this study provided a comprehensive analysis of the joint and individual effects of lipid components on MMD risk. The findings aimed to identify novel biomarkers for early diagnosis and risk stratification. Ultimately, this research sought to enhance our understanding of the role of lipid metabolism in MMD pathogenesis and inform personalized treatment strategies to improve patient outcomes.

## 2 Materials and Methods

### 2.1 Study Design and Participants

**FIGURE 1:**
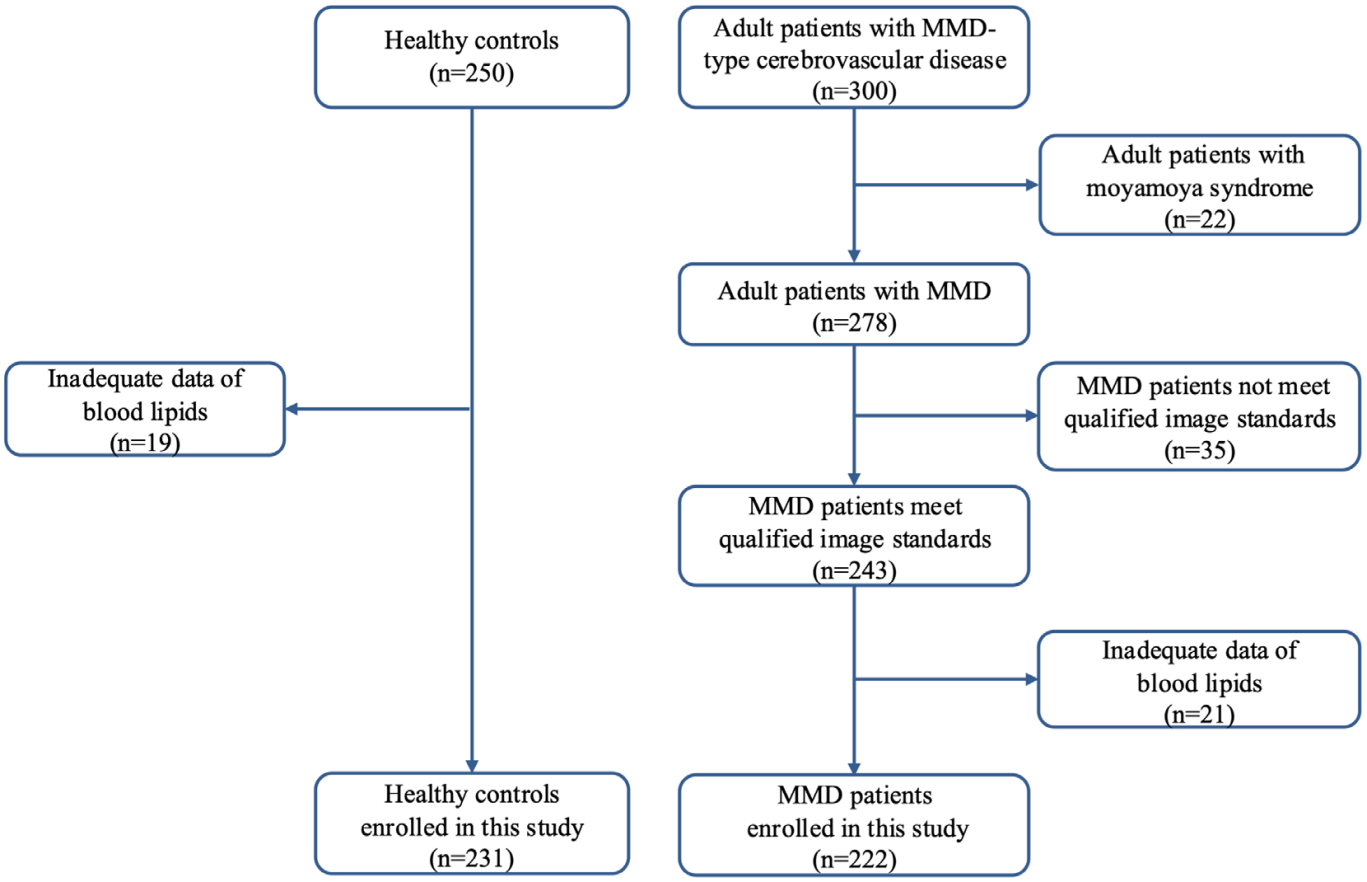
Flow diagram of the study participants. MMD, moyamoya disease.

We prospectively enrolled MMD patients at Beijing Tiantan Hospital from August 2022 to May 2024 (NCT05485870). Patients were included following the standard: (1) diagnosed with MMD according to the Japanese diagnostic criteria [13]; (2) underwent digital subtraction angiography (DSA). Patients were excluded if they: (1) were diagnosed with Moyamoya syndrome; (2) were without DSA or had poor image quality; and (3) had inadequate data of blood lipids. Overall, a total of 222 patients with MMD were included in the study (Figure 1). Besides, we recruited age‐gender‐matched healthy controls (HCs) for physical examination at Beijing Tiantan Hospital from March 2024 to April 2024. Participants were excluded if they had inadequate data of blood lipids. Eventually, 231 HCs were enrolled. The study was approved by the Ethics Committee of Beijing Tiantan Hospital (KY2022‐051‐02) and was conducted under the ethical standards of the Helsinki Declaration of 1975 (as revised in 1983). Informed consents were obtained from all participants. The reporting of this study followed the STROBE guideline (Strengthening the Reporting of Observational Studies in Epidemiology) [14].

### 2.2 Baseline Data Collection

Baseline data of MMD patients were collected including demographic characteristics (age and sex), histories of risk factors (hypertension, diabetes mellitus, hyperlipidemia, cigarette smoking, and alcohol drinking), histories of medicine (antiplatelet drugs and statins), and clinical phenotypes (ischemic, hemorrhagic, and asymptomatic). Functional neurological evaluation on admission was measured by the modified Rankin scale (mRS) score.

### 2.3 Blood Sample Collection and Genotype Sequencing

All MMD patients and HCs provided blood samples upon admission before any treatments and during physical examination, following the standardized protocol [15]. The blood samples were carefully drawn from the antecubital vein by venipuncture, and collected into ethylenediaminetetraacetic acid (EDTA) anticoagulant tubes and serum separator tubes. DNA samples were extracted from the white blood cells from peripheral blood. The *RNF213*p.R4810K variant was determined by Sanger sequencing as previously described [11]. The variants of c.388T>C and c.526C>T in *APOE* gene and c.388A>G and c.521T>C in *SLCO1B1* gene were then analyzed. Patients with *ε2*/*ε2*, *ε2*/*ε3*, and *ε3*/*ε3* genotypes were classified as none‐*ε4* carrier, and *ε3*/*ε4* or *ε4*/*ε4* genotypes were classified as *ε4* carrier [12]. Patients with *1a/*1a, *1a/*1b, *1b/*1b were grouped as type I, patients with *1a/*5, *1a/*15, *1b/*15 were grouped as type II, and patients with *5/*5, *5/*15, *15/*15 were grouped as type III [16].

### 2.4 Measurement of Circulating Lipid Profile

Serum samples were isolated from the peripheral blood and immediately stored at −80°C. Compositions of lipid profiles including TG, CHO, high‐density lipoprotein (HDL), LDL, apolipoprotein A1 (APOA1), APOA2, APOB, APOC2, APOC3, APOE, LPa, oxLDL, and sdLDL were quantified. Among them, remnant cholesterol (RC) was calculated as CHO‐HDL‐LDL. Non‐HDL was calculated as CHO‐HDL. The oxLDL levels were determined using a monoclonal antibody 4E6‐based enzyme‐linked immunosorbent assay (ELISA) kit (Xi'an GoldMag Nanobiotech Co. Ltd., China). Besides, the level of homocysteine (Hcy) and fasting blood glucose (Glu) were collected from the baseline biochemical results. The measurements were conducted in the Laboratory Diagnosis Center of Beijing Tiantan Hospital. The clinical information of participants was blinded to the technologists.

### 2.5 Classification and Assessment of Angiographic Characteristics

The angiographic features were determined using DSA in 444 hemispheres of 222 MMD patients. The assessment was independently conducted by two experienced neurosurgeons (C.Z. and Q.Z.) who were blinded to the clinical characteristics. Radiographic characteristics included Suzuki stage, posterior cerebral artery (PCA) involvement, and the collateral circulation status. Patients were dichotomized into groups of early stage (Suzuki stage 1–3) and late stage (Suzuki stage 4–6). The collateral circulation status was classified as grade I (scores 1–4), grade II (scores 5–8), and grade III (scores 9–12) [17].

### 2.6 Statistical Analysis

The baseline characteristics and levels of lipid profile were compared between groups of MMD patients and healthy controls. Categorical data were presented as frequencies with percentages and analyzed by *χ* ^2^ test or Fisher exact test. Continuous variables were expressed as mean with standard deviation (SD) or median with interquartile range (IQR) and analyzed using *t*‐tests or Mann–Whitney *U* tests. Receiver operating characteristic (ROC) curves were constructed to screen the significant lipid biomarkers associated with the risk of MMD according to the area under the curve (AUC). Logistic regression models were used to analyze the relationship between oxLDL and MMD as well as its subtypes. Odds ratio (OR) with 95% confidence interval (CI) were calculated. Covariates of age and sex were adjusted in Model 1, whereas TG, CHO, APOA1, APOA2, APOC2, APOC3, APOE, Hcy, sdLDL, RC, LPa, Glu, and non‐HDL were further adjusted in Model 2. The weighted quantile sum (WQS) model was applied to evaluate the combined effect of the lipid mixture on MMD risk [18]. This approach constructed a weighted index reflecting the relative contribution of each lipid biomarker, with individual weights ranging from 0 to 1 and summing to 1. In our analysis, we performed bootstrapping with 100 iterations to stabilize the WQS estimates while adjusting for age, hypertension, diabetes, and history of statin use. In parallel, Spearman's rank correlation was employed to assess pairwise relationships among the lipid components. To further disentangle potential nonlinear and interaction effects within the lipid mixture, we used Bayesian kernel machine regression (BKMR), a semi‐parametric framework capable of partitioning the joint effect into main and interactive components [19]. Univariate and bivariate exposure‐response functions were generated to examine these main and interaction effects in greater detail. Covariate selection differed across models to align with their analytic objectives: logistic regression adjusted for multiple lipid parameters to isolate the independent effect of oxLDL, whereas WQS and BKMR adjusted only for nonlipid confounders (age, hypertension, diabetes, and statin use) to avoid overadjustment within mixture models. The statistical analyses were conducted using SPSS (version 26.0, IBM Corporation, Armonk, NY, USA) and R (version 4.3.2, R Development Core Team). A two‐tailed *p* < 0.05 was considered statistical significance.

## 3 Results

A total of 222 patients with MMD and 231 HCs were enrolled in the study.

### 3.1 Baseline Characteristics of the Participants

Demographic characteristics of 222 MMD patients (mean age = 41.0 ± 19.0 years; female/male = 1.22/1) and 231 HCs (mean age = 35.0 ± 13.0 years; female/male = 1.02/1) were compared (Table S1). The baseline features of MMD patients were summarized (Table S2).

### 3.2 Comparison of Lipids Levels Between Different Groups

**FIGURE 2:**
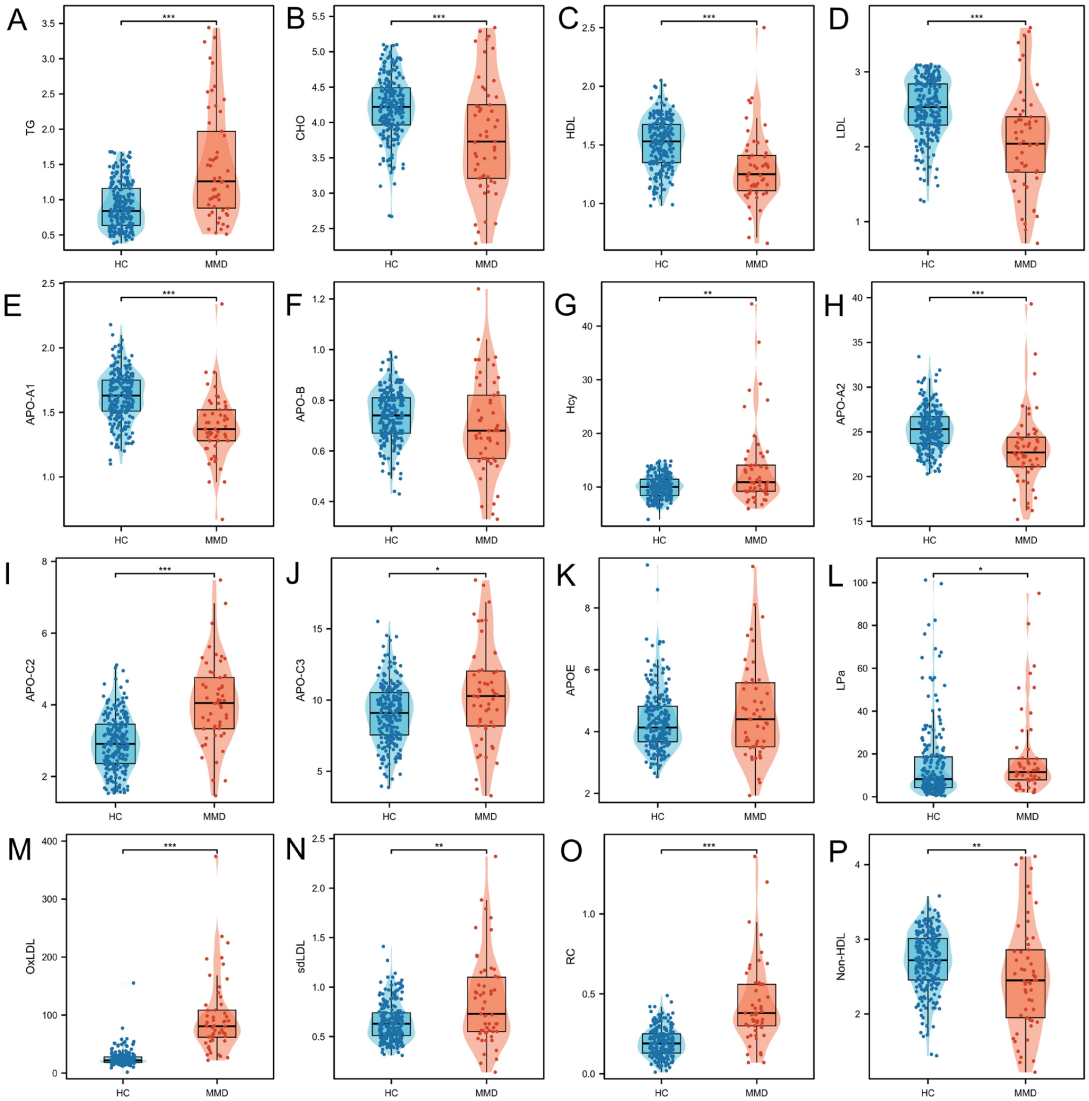
Comparisons of lipids profile between MMD patients and HCs. (A–P) Comparisons of TG, CHO, HDL, LDL, APOA1, APOB, Hcy, APOA2, APOC2, APOC3, APOE, LPa, oxLDL, sdLDL, RC, and non‐HDL between MMD patients and HCs. *p < 0.05; **p < 0.01; ***p < 0.001. APOA1, apolipoprotein A1; APOA2, apolipoprotein A2; APOB, apolipoprotein B; APOC2, apolipoprotein C2; APOC3, apolipoprotein C3; APOE, apolipoprotein E; CHO, cholesterol; HC, healthy control; Hcy, homocysteine; HDL, high‐density lipoprotein; LDL, low‐density lipoprotein; LPa, lipoprotein(a); MMD, moyamoya disease; non‐HDL, none high‐density lipoprotein; oxLDL, oxidized low‐density lipoprotein; RC, remnant cholesterol; sdLDL, small dense low‐density lipoprotein; TG, triglyceride.

Levels of lipid profiles and Hcy were compared between groups of MMD patients and HCs. MMD patients showed significantly higher levels of TG, APOC2, APOC3, LPa, oxLDL, sdLDL, RC, and Hcy, and lower levels of CHO, HDL, LDL, APOA1, APOA2, and Non‐HDL (*p* < 0.05 for all comparisons) (Figure 2). MMD patients with a history of statins had significantly lower levels of CHO, LDL, APOA1, APOA2, and non‐HDL, whereas they had higher levels of TG, APOC2, APOC3, APOE, oxLDL, sdLDL, RC, and Hcy, compared with HCs (*p* < 0.05 for all comparisons) (Figure S1). MMD patients without a history of statins had significantly higher levels of TG, APOC2, APOC3, APOE, LPa, oxLDL, sdLDL, and RC, compared with HCs (*p* < 0.05 for all comparisons). Additionally, MMD patients carrying the *ApoE ε4* genotype exhibited significantly increased levels of CHO, LDL, APOB, oxLDL, and sdLDL, compared with non‐*ε4* genotype patients (Figure S2). MMD patients with *RNF213*p.R4810K variant had elevated levels of HDL, APOA1, and RC, compared to wild‐type patients (Figure S3). Regarding the clinical manifestations and angiographic stage, levels of CHO, HDL, LDL, APOB, and non‐HDL in hemorrhagic patients were higher than those in ischemic patients (Figure S4), whereas no significant difference was demonstrated between groups of Suzuki 1–3 and 4–6 (Figure S5).

### 3.3 Associations of oxLDL With the Risk of MMD and Subtypes

**FIGURE 3:**
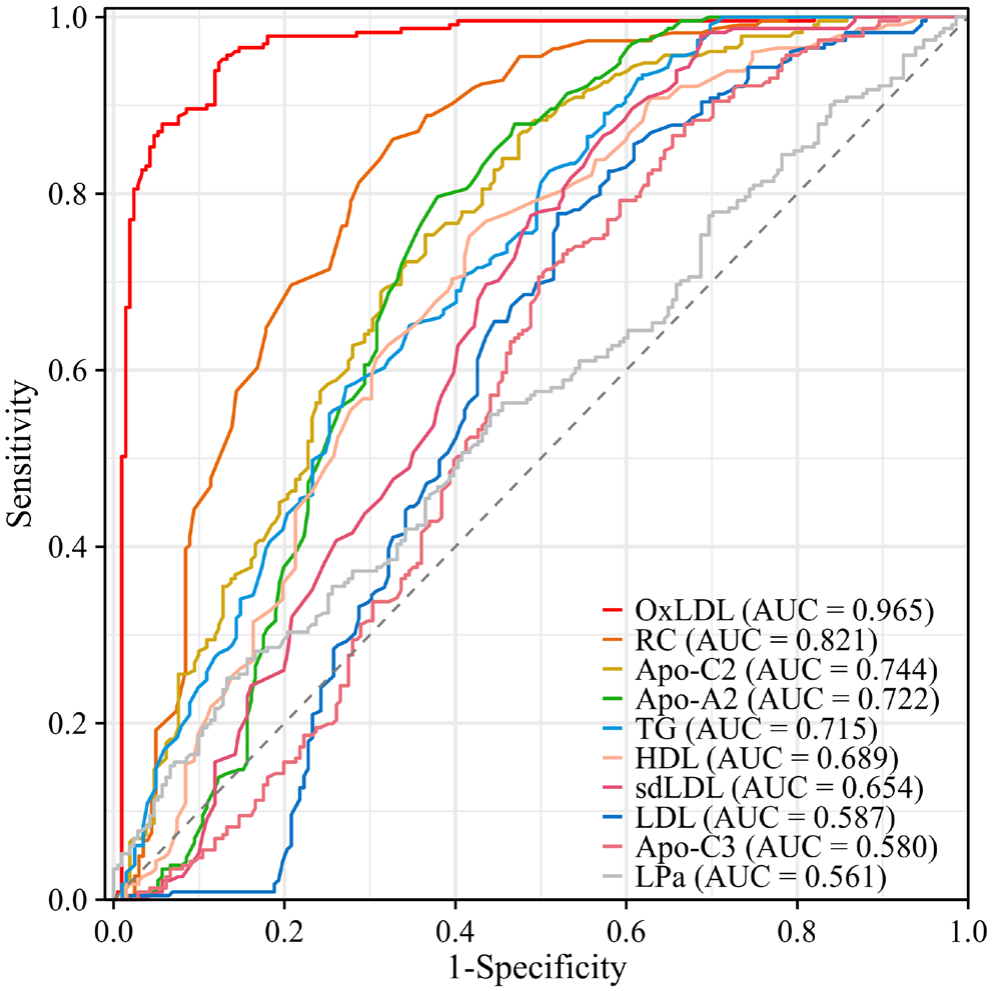
ROC curves with AUC of different lipids for predicting the risk of MMD. APOA2, apolipoprotein A2; APOC2, apolipoprotein C2; APOC3, apolipoprotein C3; HDL, high‐density lipoprotein; LDL, low‐density lipoprotein; LPa, lipoprotein(a); oxLDL, oxidized low‐density lipoprotein; RC, remnant cholesterol; sdLDL, small dense low‐density lipoprotein; TG, triglyceride.

**FIGURE 4:**
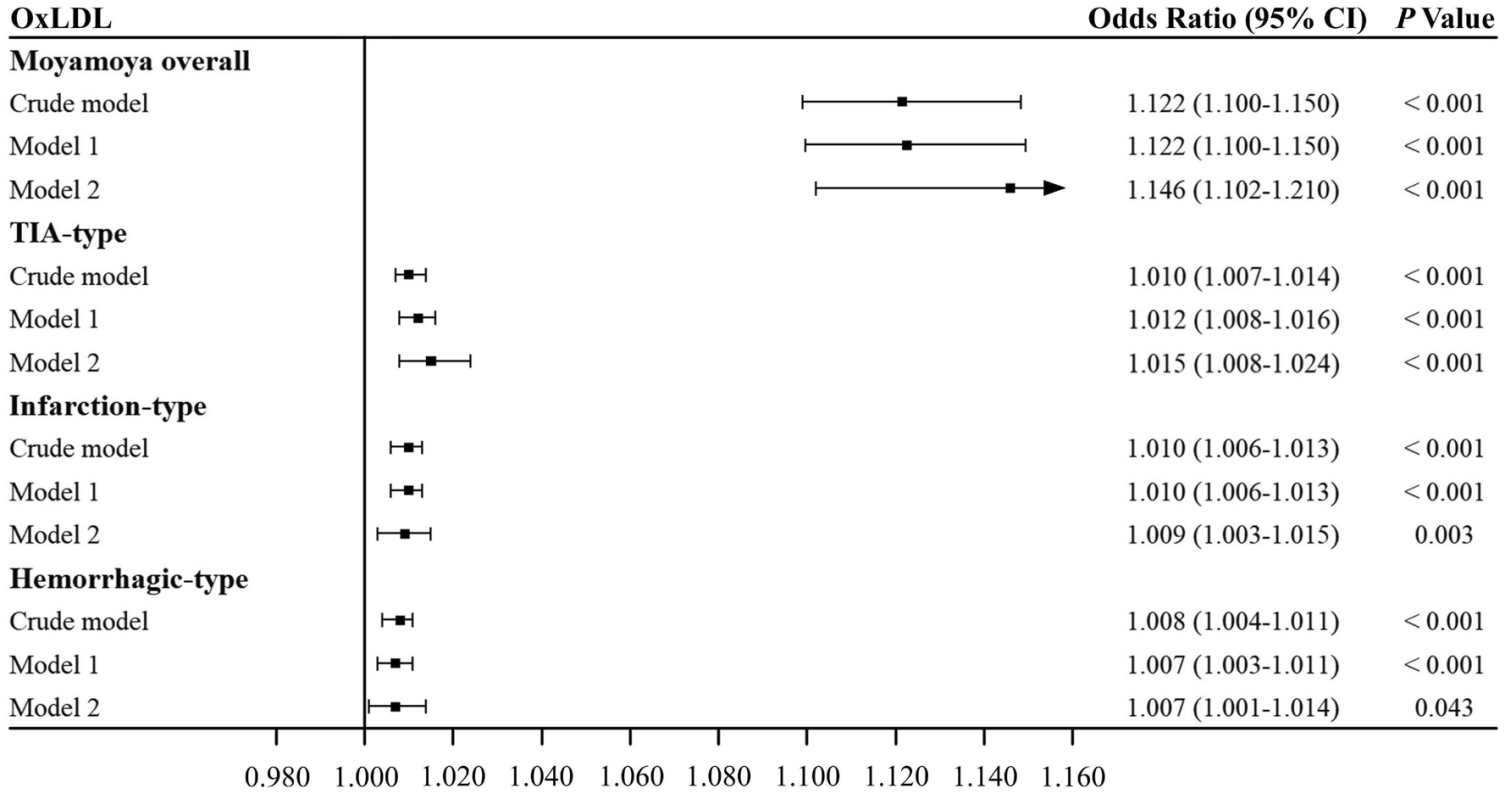
Associations of oxLDL with the risk of MMD and its subtypes. OxLDL was significantly related to a higher risk of MMD and three clinical subtypes. Model 1, adjusted for age and sex. Model 2, further adjusted for TG, CHO, APOA1, APOA2, APOC2, APOC3, APOE, Hcy, sdLDL, RC, LPa, Glu, and non‐HDL. CI, confident interval; oxLDL, oxidized low‐density lipoprotein; TIA, transient ischemic attack.

ROC curves with AUC values for various lipid profiles related to the risk of MMD were presented in Figure 3. Among these, OxLDL exhibited the highest AUC of 0.965, indicating superior diagnostic power compared to the other lipid profiles. We constructed both univariate and multivariate logistic regression models to analyze the association between oxLDL and the risk of MMD as well as its subtypes (Figure 4). In the univariate analyses, oxLDL was found to be significantly associated with the risk of MMD and its three subtypes. When we included covariates in the multivariate analyses (Model 2), oxLDL remained independently associated with the risk of MMD and all three subtypes: MMD overall (OR 1.146, 95% CI 1.102–1.210, *p* < 0.001), transient ischemic attack (TIA)‐type (OR 1.015, 95% CI 1.008–1.024, *p* < 0.001), infarction‐type (OR 1.009, 95% CI 1.003–1.015, *p* = 0.003), and hemorrhagic‐type (OR 1.007, 95% CI 1.001–1.014, *p* = 0.043).

### 3.4 Association of Lipids Co‐Exposure With the Risk of MMD

**FIGURE 5:**
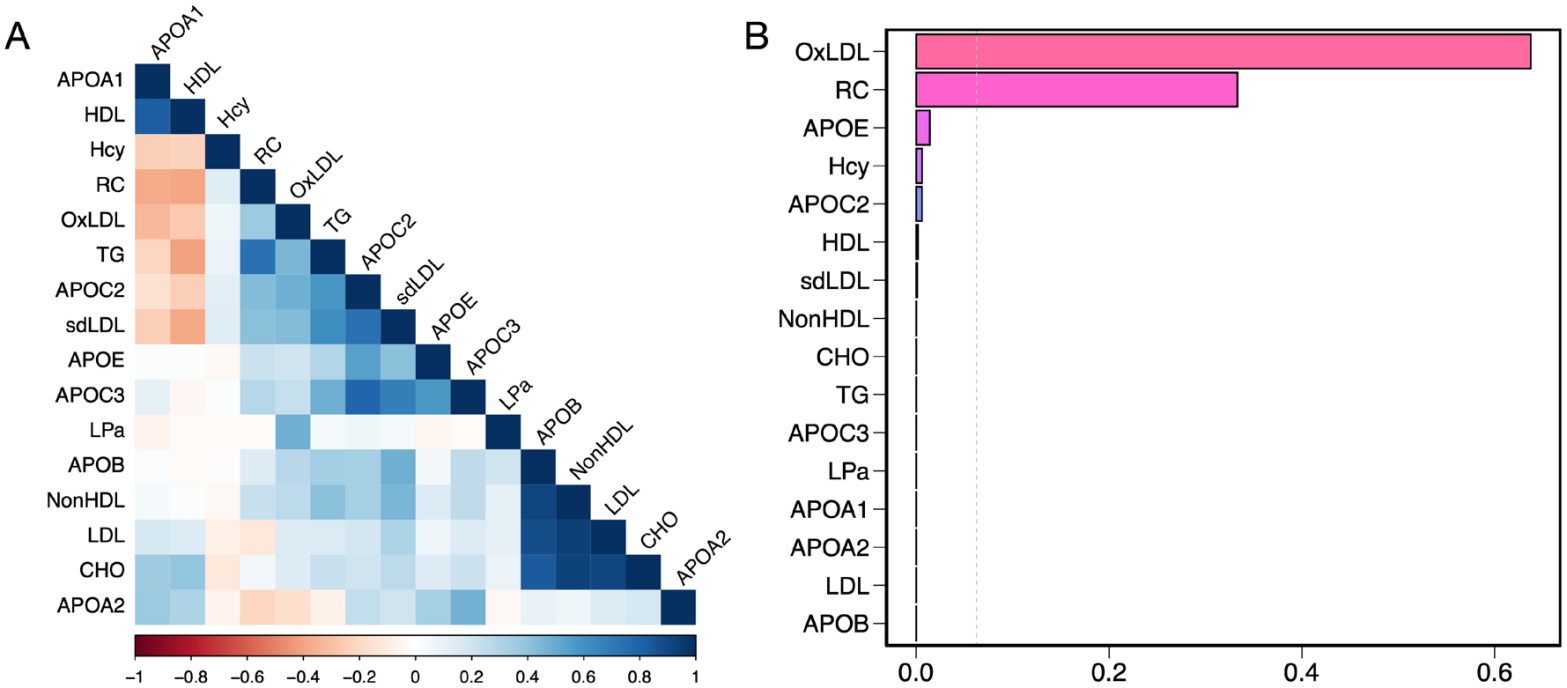
Association between lipids co‐exposure and the risk of MMD determined by the WQS regression model. (A) Pairwise relationships among the lipid components. (B) Association of lipids co‐exposure with the risk of MMD. Estimated weights of each specific lipid among the joint effect on MMD risk. The WQS regression model was adjusted for age, hypertension, diabetes, and history of statin use. APOA1, apolipoprotein A1; APOA2, apolipoprotein A2; APOB, apolipoprotein B; APOC2, apolipoprotein C2; APOC3, apolipoprotein C3; APOE, apolipoprotein E; CHO, cholesterol; Hcy, homocysteine; HDL, high‐density lipoprotein; LDL, low‐density lipoprotein; LPa, lipoprotein(a); non‐HDL, none high‐density lipoprotein; oxLDL, oxidized low‐density lipoprotein; RC, remnant cholesterol; sdLDL, small dense low‐density lipoprotein; TG, triglyceride.

The correlation analysis revealed a strong positive relationship between APOA1 and HDL (Figure 5A), consistent with their biological interaction. A strong correlation was also observed between OxLDL and sdLDL, suggesting the potential coaction of these lipoprotein subtypes in pathological processes. Furthermore, we employed the WQS model to explore the association between lipid profiles and the risk of MMD (Figure 5B). The model was designed to assess positive associations while accounting for age, hypertension, diabetes, and history of statins. The analysis revealed that the WQS index of combined lipid exposure was significantly associated with an increased risk of MMD (OR = 1.465, 95% CI 1.405–1.527, *p* < 0.001). Among the lipid components, oxLDL (0.64) carried the highest weight in the index, followed by RC (0.33), highlighting the substantial contribution of oxLDL to the positive association between the WQS index and MMD risk.

**FIGURE 6:**
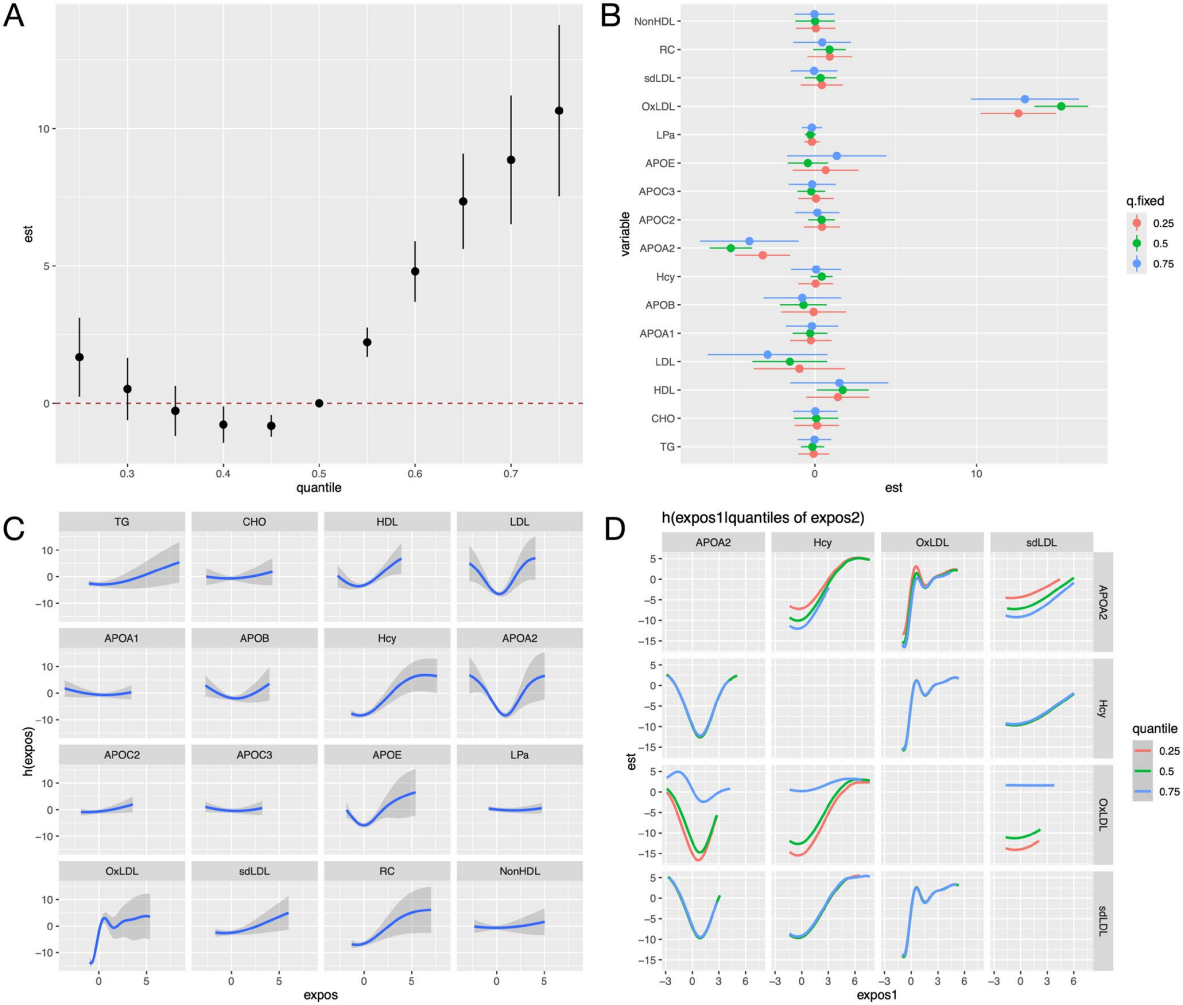
Association between mixed and individual lipids exposure and the risk of MMD determined by the BKMR model. (A) The joint effect of the lipid mixture on the risk of MMD. All 16 components were analyzed as a mixture. The results indicated a significant increase in the risk of MMD when the lipid mixture was in the 50th to 75th percentile compared to the median. (B) The individual effect of each lipid on the risk of MMD, with other lipids fixed at the 25th, 50th, and 75th percentiles. (C) Univariate exposure‐response functions for each lipid associated with the risk of MMD, with other lipids fixed at the median. (D) Bivariate associations between different lipid substances and the risk of MMD, with other lipids fixed at the median. The BKMR models were adjusted for age, hypertension, diabetes, and history of statin use. APOA1, apolipoprotein A1; APOA2, apolipoprotein A2; APOB, apolipoprotein B; APOC2, apolipoprotein C2; APOC3, apolipoprotein C3; APOE, apolipoprotein E; CHO, cholesterol; Hcy, homocysteine; HDL, high‐density lipoprotein; LDL, low‐density lipoprotein; LPa, lipoprotein(a); non‐HDL, none high‐density lipoprotein; oxLDL, oxidized low‐density lipoprotein; RC, remnant cholesterol; sdLDL, small dense low‐density lipoprotein; TG, triglyceride.

To further explore the potential interactions among various lipids and their collective impact on MMD, a BKMR model was employed to evaluate the effect of lipid co‐exposure on the risk of MMD, adjusting for age, hypertension, diabetes, and history of statins. The cumulative effect of the lipid mixture on the risk of MMD was illustrated (Figure 6A), where all 16 components were analyzed as a mixture. The results indicated a significant increase in the risk of MMD when the lipid mixture was in the 50th to 75th percentile, compared to the median. Furthermore, when other lipid components were fixed at the 25th, 50th, and 75th percentiles, oxLDL exhibited a significant positive association with the risk of MMD (Figure 6B). In addition, univariate analyses were conducted to examine the individual associations between lipid components and the risk of MMD (Figure 6C). Elevated levels of TG, oxLDL, sdLDL, RC, and Hcy were positively correlated with increased MMD risk, whereas components such as HDL, LDL, APOA2, and APOE demonstrated nonlinear trends. The interaction analysis between different lipid substances and the risk of MMD was demonstrated in Figure 6D. Notably, the interaction between oxLDL and Hcy revealed that at higher percentiles (≥ 75th), their joint exposure was associated with an amplified increase in MMD risk compared to median or lower percentiles, suggesting a synergistic effect.

## 4 Discussion

This study provided a comprehensive analysis of the lipid profile in MMD and explored the potential role of lipid co‐exposure in disease risk. Notably, it highlighted the novel discovery of oxLDL as a significant biomarker positively associated with MMD risk. By leveraging advanced statistical models, we systematically evaluated the joint and individual effects of various lipid components on the risk of MMD.

Our findings underscored the multifaceted roles that dysregulated lipid metabolism may contribute to the pathogenesis of MMD. Previous studies have largely focused on the genetic predisposition and hemodynamic changes that underly MMD [20, 21], yet our data suggested that specific lipid subclasses and their oxidative modifications could contribute substantially to the onset and subtypes of the disease. Consistently, accumulating evidence indicated that dyslipidemia was a prevalent feature of MMD. Several studies have reported elevated levels of CHO, TG, and Lpa in patients with MMD, as well as aberrant expression patterns revealed by high‐throughput lipidomic analyses in peripheral blood [7, 8, 22]. Notably, a recent study employing advanced cerebrovascular lipidomics on intracranial and extracranial cerebrovascular samples from patients with MMD and intracranial atherosclerotic disease has expanded our understanding of the intricate lipid networks implicated in these distinct vasculopathies [6]. By uncovering novel lipid biomarkers involved in inflammatory cascades, endothelial dysfunction, and vascular remodeling, this approach reinforces the notion that an abnormal lipid milieu in MMD is not limited to peripheral circulation but extends to the cerebral vasculature itself. These findings suggested that localized cerebrovascular lipid abnormalities may occur independently of systemic lipid profiles, underscoring the distinct metabolic features of MMD vasculopathy. Additionally, our research team has previously documented increased lipid levels, especially in certain apolipoproteins in the peripheral blood of MMD patients [7], aligning with the notion that dyslipidemia may accelerate or exacerbate the disease process. Integrating these cerebrovascular lipidomics findings with peripheral lipid profiles could yield a more holistic picture of disease mechanisms and further pinpoint therapeutic targets.

The identification of oxLDL as a significant biomarker in MMD represented a pivotal finding in this study. OxLDL has been recognized as a key pro‐inflammatory mediator that promotes endothelial dysfunction, arterial wall remodeling, and plaque progression in various vascular disorders [9]. Although MMD is not traditionally characterized as an atherosclerotic disease, pathologic changes involving the proliferation of the intima and media as well as infiltration of inflammatory cells bear resemblance to arterial remodeling observed in other vasculopathy [3]. The immunopathological assessments have confirmed the infiltration of macrophages and T cells within MMD‐affected vessels [23], and specimens acquired during bypass operations offer novel insights into the dynamics of immune infiltration [24, 25], thereby reinforcing the theory that an immune‐mediated pathological process plays a pivotal role in arterial remodeling of MMD. It is plausible that oxLDL, by triggering endothelial injury and stimulating smooth muscle cell migration, further destabilizes the cerebrovasculature in individuals with underlying genetic susceptibility. Nevertheless, oxLDL should be regarded as a potential biomarker rather than definitive evidence of causality in MMD, pending further experimental validation. It should be noted that oxLDL levels in this study were measured using a 4E6 monoclonal antibody‐based ELISA, which specifically detected oxidatively modified epitopes on apoB‐100, but did not differentiate degrees of oxidation, potentially underestimating the heterogeneity of oxLDL species involved in MMD pathophysiology. In addition, the strong correlation we observed between oxLDL and sdLDL echoes findings in atherosclerotic contexts, where these small, dense LDL particles are both more prone to oxidation and more adept at penetrating the arterial intima [26]. Such interplay may potentiate a vicious cycle of oxidative stress and local inflammation, culminating in the progressive luminal narrowing characteristic of MMD. Although oxLDL was significantly elevated in MMD patients compared to healthy controls in our study, it is important to note that elevated oxLDL levels are also observed in atherosclerosis diseases [27]. Thus, the specificity of oxLDL as a biomarker for MMD requires further validation in future comparative studies.

The application of mixture models, including WQS and BKMR, revealed that the cumulative co‐exposure to multiple lipid constituents was significantly associated with an increased risk of MMD. Conventional epidemiologic methods often treat individual biomarkers in isolation, potentially underestimating the complex synergy that drives disease. By capturing a weighted summary index, we identified oxLDL and RC as major contributors to the shared variance underlying the lipid burden. This suggested that neither a single lipoprotein fraction nor total cholesterol alone is sufficient to characterize the risk of dyslipidemia in MMD. Instead, the interplay between highly atherogenic lipoprotein remnants and oxidatively modified LDL particles may provoke more profound endothelial derangements. Our BKMR analysis further bolstered these observations, showing that elevated percentiles of lipids, especially oxLDL, portend an increased probability of MMD. Notably, Hcy also displayed potential synergy with oxLDL at higher concentration ranges, a biologically plausible interaction given that both oxidative stress and Hcy‐induced endothelial toxicity can converge to impair vascular integrity [28]. Recent evidence suggested that this interaction may involve shared pathological pathways, such as NF‐κB signaling activation and enhanced reactive oxygen species (ROS) production, ultimately exacerbating endothelial dysfunction and inflammatory responses central to MMD vascular remodeling [29, 30]. Moreover, our previous research indicated that peripheral Hcy levels in MMD patients were higher compared to those in controls, thereby underscoring hyperhomocysteinemia as another potentially modifiable factor contributing to MMD pathogenesis [7]. Future mechanistic research should delve into whether combined interventions targeting multiple lipids and Hcy might achieve better vascular protection than addressing any single factor alone.

In parallel, our observation that *APOE ε4* carriers displayed significantly higher levels of oxLDL, sdLDL, and LDL underscored the interplay between *APOE* polymorphisms and lipid metabolism in MMD. The *APOE* locus is a well‐established determinant of plasma lipoprotein profiles; *ε4* is associated with elevated LDL, increased oxidation susceptibility, and heightened atherosclerosis risk in the general population [31]. While MMD is distinct from typical atherosclerosis, an *APOE ε4*‐driven propensity toward more atherogenic lipoprotein subclasses could conceivably intersect with the unique vascular milieu of MMD, exacerbating endothelial dysfunction and pathological neovascularization. Interestingly, a prior study of 86 MMD patients and 83 healthy controls detected no significant differences in the overall distribution of *APOE* genotypes between the two cohorts. However, among the MMD group, individuals carrying the *ε2* or *ε4* alleles exhibited a higher incidence of cerebral microbleeds—an established biomarker of cerebral small vessel disease—suggesting that *APOE* variants may be linked to specific disease phenotypes or increased disease severity in MMD [12]. The nonlinear relationship observed between APOE lipid levels and MMD risk in our BKMR analysis aligned with previous reports indicating a complex biological effect of APOE, where moderate levels confer vascular protection, whereas excessively low or high concentrations, particularly associated with the *APOE* ε4 isoform, may exacerbate endothelial injury through oxidative and inflammatory pathways [32]. As such, routine genotyping of *APOE* may be warranted in future studies aiming to stratify MMD risk or to personalize lipid‐lowering therapies in these patients.

Our results also demonstrated the impact of statin therapy on the lipid profiles in MMD. Although statins are standard‐of‐care for dyslipidemia, their role in MMD remains uncertain [33]. In the present study, patients with a history of statins did exhibit lower LDL, CHO, and certain apolipoproteins, which was consistent with the known lipid‐lowering action of these medications. Intriguingly, statin users still maintained elevated oxLDL and sdLDL levels relative to healthy controls, and they also demonstrated increases in RC and Hcy. These findings raised several possibilities. One scenario is that standard statin regimens may be insufficient to normalize atherogenic lipoprotein remnants or address the oxidative stress pathways that fuel oxLDL formation [10]. Alternatively, patients prescribed statins might have more severe baseline dyslipidemia or systemic inflammation, rendering them less responsive to conventional lipid‐lowering approaches. Notably, a recent large‐scale cohort study from Korea found that statin therapy may reduce long‐term stroke risk in MMD, while research in China indicated that statin use could facilitate postoperative angiogenesis in MMD [34, 35]. Although high‐level randomized controlled trials are not yet available, these observations collectively suggested that statins may confer certain benefits in MMD management. Nonetheless, statins do not address all lipid abnormalities, particularly oxLDL. These findings underscored the need for adjunctive interventions aimed at mitigating lipoprotein oxidation or clearing remnant particles. Overall, our data suggested that while statins can partially correct conventional lipid abnormalities in MMD, more comprehensive metabolic control likely requires targeted therapies beyond statin monotherapy.

It is important to acknowledge several limitations in our study. First, although our case–control design provided robust cross‐sectional data, we were unable to make definitive inferences about causality. Disentangling whether these lipid abnormalities preceded or followed the vascular remodeling intrinsic to MMD required longitudinal data. Second, our single‐center enrollment and relatively limited sample size, despite being one of the larger studies of its kind, may not capture the full clinical heterogeneity across different ethnicities or geographic regions. Third, although we applied advanced statistical modeling techniques, residual confounding from unmeasured variables, such as lifestyle factors (body mass index, diet, and physical activity), genetic modifiers beyond *RNF213* and *APOE*, or unknown inflammatory conditions, remained possible. Fourth, we did not assess potential functional differences in HDL subfractions or the role of oxidative stress biomarkers, which may also be relevant mechanistic factors. Fifth, although our findings suggested a strong association between oxLDL and MMD, this link was currently based on observational data. Experimental validations are essential to confirm a causal role for oxLDL in promoting cerebrovascular pathology in MMD. Finally, our study did not address potential therapeutic interventions beyond statins, nor did it explore whether intensifying statin therapy or combining it with agents that specifically lower oxLDL could offer superior vascular protection. Future randomized trials designed to clarify these issues would provide valuable direction for management strategies.

## 5 Conclusions

In summary, our comprehensive analysis delineated a complex dyslipidemic landscape in MMD. The identification of oxLDL as a key biomarker and the demonstration of cumulative lipid effects underscored the importance of lipid metabolism in MMD. Further mechanistic investigations are necessary to elucidate how these specialized lipid fractions trigger the onset and progression of MMD, and may ultimately pave the way for novel diagnostic and therapeutic strategies.

## Author Contributions

Study concept and design: Qian Zhang and Chaofan Zeng. Provision of study materials or patients: Qian Zhang, Guojun Zhang, and Jizong Zhao. Collection and assembly of data: Chaofan Zeng, Haoyuan Chen, Youyuan Bao, Xudong Sun, Fanbo Meng, Yimeng Xue, Yunhao Cui, Qianjun Zhao, Jing Zhang, and Hao Li. Data analysis and interpretation: Chaofan Zeng, Haoyuan Chen, and Jie Liu. Manuscript writing: Chaofan Zeng, and Haoyuan Chen. Manuscript revision: Dong Zhang, Rong Wang, Yan Zhang, and Qian Zhang. Final approval of manuscript: All authors.

## Disclosure

The authors have nothing to report.

## Conflicts of Interest

The authors declare no conflicts of interest.

## Supporting information


Appendix S1

## Data Availability

The data that support the findings of this study are available from the corresponding authors on reasonable request.
