# Supplementary material for: Unraveling the Dyslipidemic Landscape in Moyamoya Disease: OxLDL as a Key Biomarker
Source: CNS Neurosci Ther. 2025 May 14;31(5):e70441. doi: 10.1111/cns.70441 (PMC12076191; doi:10.1111/cns.70441)
Supplement: Supplementary file 1 — Appendix S1 [file CNS-31-e70441-s001.pdf]

SUPPLEMENTAL MATERIAL

Unraveling the Dyslipidemic Landscape in Moyamoya Disease: OxLDL as a Key Biomarker

Chaofan Zeng[1-4,†], MD, Haoyuan Chen[1-4,†], MD, Jie Liu[5], MD, Youyuan Bao[1-4], MD, Xudong Sun[1-4], MD, Fanbo Meng[1-4], MD, Yimeng Xue[6], MD, Yunhao Cui[1-4], MD, Qianjun Zhao[1-4], MD, Jing Zhang[7], MD, Hao Li[1-4], MD, Dong Zhang[8], MD, Rong Wang[1-4], MD, Yan Zhang[1-4], MD, Guojun Zhang[5], MD[‡], Jizong Zhao[1-4], MD[‡], Qian Zhang[1-4], MD[‡]

[1]Department of Neurosurgery, Beijing Tiantan Hospital, Capital Medical University, Beijing, China

[2]China National Clinical Research Center for Neurological Diseases, Beijing, China

[3]Center of Stroke, Beijing Institute for Brain Disorders, Beijing, China

[4]Beijing Key Laboratory of Translational Medicine for Cerebrovascular Disease, Beijing, China

[5]Department of Clinical Diagnosis, Laboratory of Beijing Tiantan Hospital, Capital Medical University, Beijing, China

[6]Department of Neuropathology, Beijing Neurosurgical Institute, Beijing, China

[7]Capital Medical University, Beijing, China

[8]Department of Neurosurgery, Beijing Hospital, National Center of Gerontology, Beijing, China

[‡]**Corresponding Author:**

Qian Zhang, MD, Department of Neurosurgery, Beijing Tiantan Hospital, Capital Medical University, No. 119 South 4th Ring West Road, Fengtai District, 100070, Beijing, China

E-mail: zhangqianchina@yahoo.com

Jizong Zhao, MD, Department of Neurosurgery, Beijing Tiantan Hospital, Capital Medical University, No. 119 South 4th Ring West Road, Fengtai District, 100070, Beijing, China

E-mail: zhaojizong@bjtth.org

Guojun Zhang, MD, Department of Clinical Diagnosis, Laboratory of Beijing Tiantan Hospital, Capital Medical University, No. 119 South 4th Ring West Road, Fengtai District, 100070, Beijing, China

E-mail: guojun.zhang@ccmu.edu.cn

†**These authors contributed equally to this work.**

Table S1. Baseline characteristics of HCs and MMD patients.

Variables	HCs (n=231)	MMD (n=222)	*P* value
Age, median (IQR)	35 (13.0)	41 (19.0)	0.227
Sex, female, n (%)	117 (50.6)	122 (55.0)	0.416

HCs, healthy controls; MMD, moyamoya disease; IQR, interquartile range.

*P < 0.05, significant difference.

Table S2. Baseline characteristics of MMD patients.

Variables	MMD (n=222)
Age, years, median (IQR)	41.00 (30.00, 49.00)
Sex, female, n (%)	122 (55.00)
BMI, mean (SD)	24.90 (4.56)
History of risk factors, n (%)	
Hypertension	76 (34.20)
Diabetes Mellitus	29 (13.10)
Coronary heart disease	8 (3.60)
Dyslipidemia	21 (9.50)
Cigarette smoking	32 (14.40)
Alcohol drinking	19 (8.60)
History of medication, n (%)	
Antiplatelet drugs	38 (17.12)
Statins	53 (23.90)
Clinical phenotypes, n (%)	
Ischemic	155 (69.80)
Hemorrhagic	52 (23.42)
Asymptomatic	15 (6.70)
Admission mRS score, mean (SD)	0.50 (0.73)
Genotype, n (%)	
ApoE genotype†	
ε4	39 (17.50)
Non-ε4	153 (68.90)
SLCO1B1 genotype†	
I	159 (71.60)
II	28 (12.60)
III	5 (2.20)
*RNF213*p.R4810K variant	53 (23.90)

Angiographic characteristics, n (%)	
Suzuki stage‡	
I-III	327 (73.65)
IV-VI	117 (26.35)
PCA involvement	58 (26.10)
Collateral circulation	
Grade I (1-4)	19 (8.60)
Grade II (5-8)	178 (80.20)
Grade III (9-12)	25 (11.30)

MMD, moyamoya disease; IQR, interquartile range; BMI, body mass index; SD, standard deviation; mRS, modified Rankin scale; PCA, posterior circulation artery.

†A total of 30 MMD patients were lack of *ApoE* and *SLCO1B1* genotype.

‡Features of 444 hemispheres in 222 MMD patients.

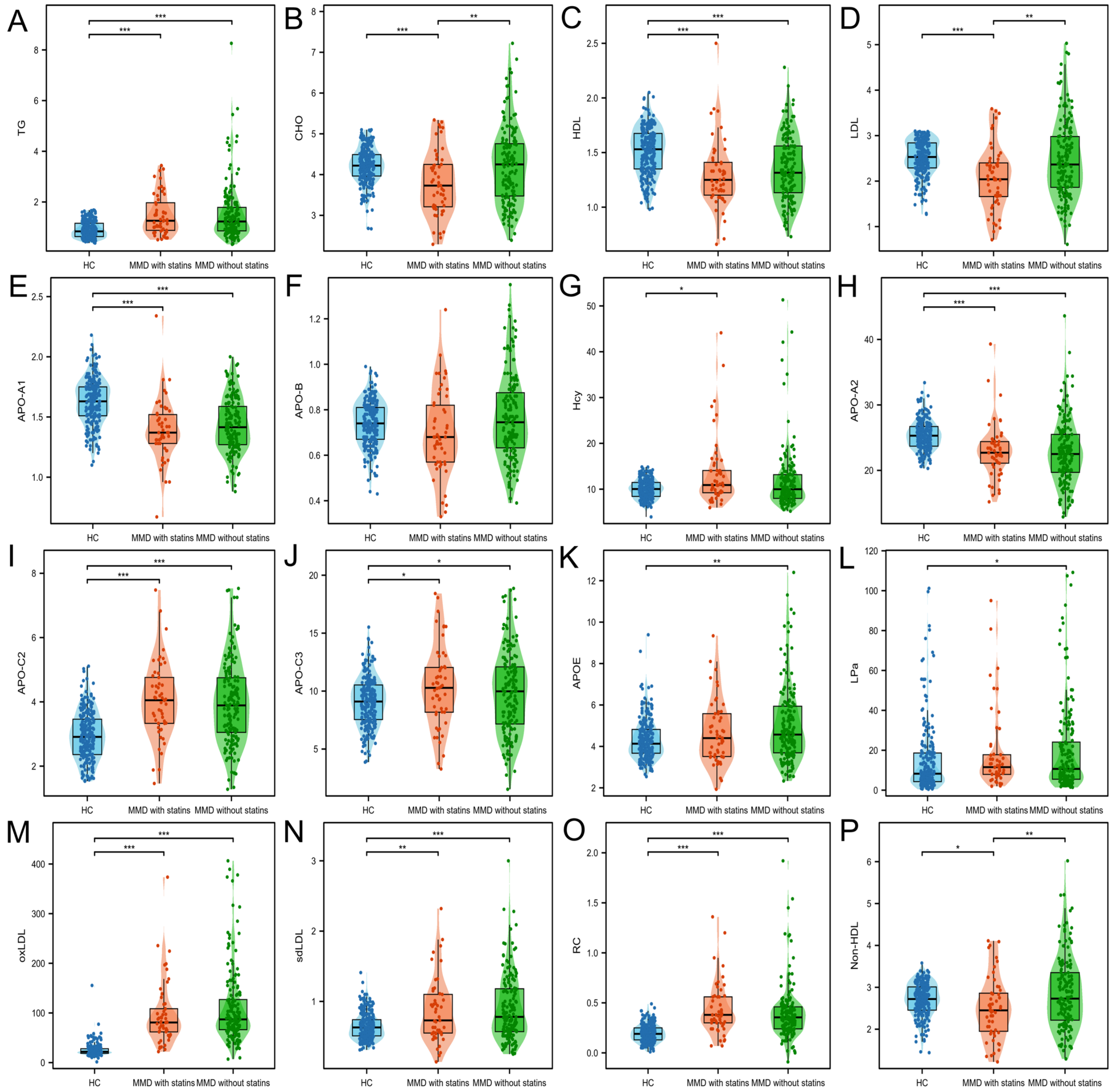

Figure S1. Comparisons of lipids profile between MMD patients with or without statins and HCs. A-P. Comparisons of TG, CHO, HDL, LDL, APOA1, APOB, Hcy, APOA2, APOC2, APOC3, APOE, LPa, oxLDL, sdLDL, RC, and non-HDL between MMD patients with statin or without statins and HCs.

*P < 0.05; **P < 0.01; *** P < 0.001.

MMD, moyamoya disease; HC, healthy control; TG, triglyceride; CHO, cholesterol; HDL, high-density lipoprotein; LDL, low-density lipoprotein; APOA1, apolipoprotein A1; APOB, apolipoprotein B; Hcy, homocysteine; APOA2, apolipoprotein A2; APOC2, apolipoprotein C2; APOC3, apolipoprotein C3; APOE, apolipoprotein E; LPa, lipoprotein(a); oxLDL, oxidized low-density

lipoprotein; sdLDL, small dense low-density lipoprotein; RC, remnant cholesterol; non-HDL, none high-density lipoprotein.

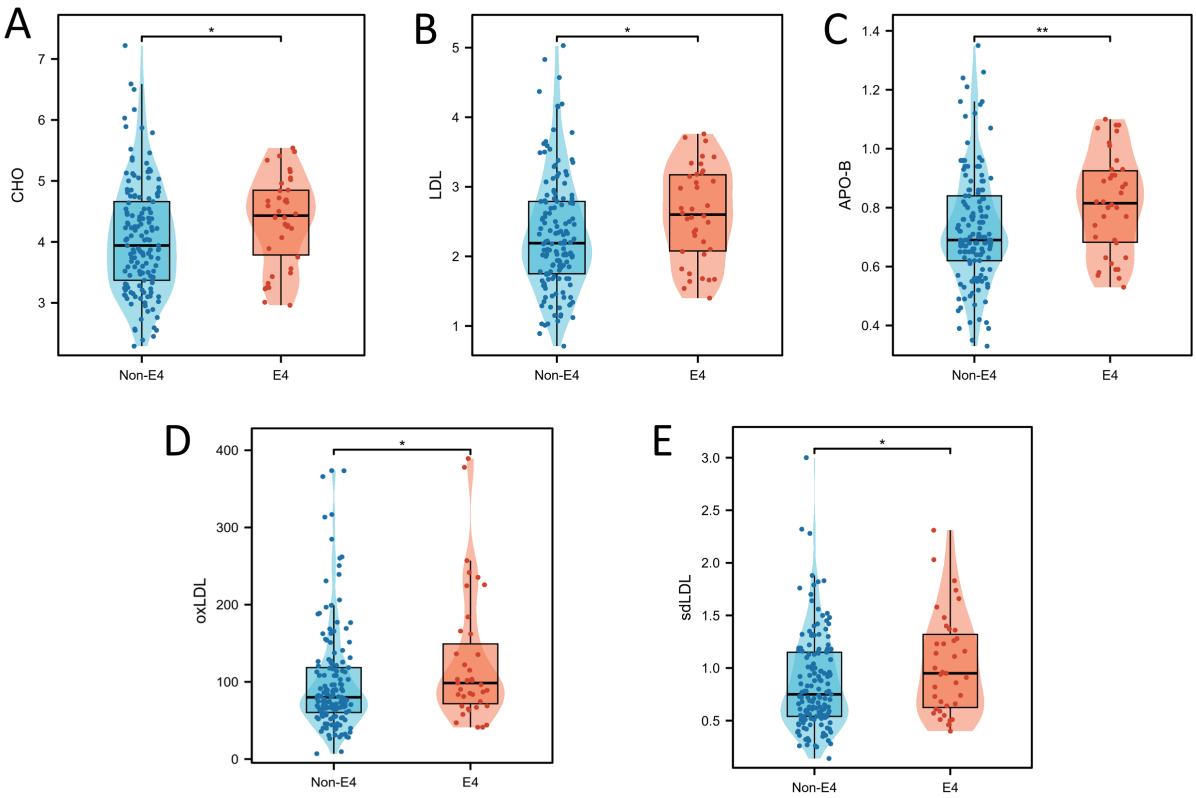

Figure S2. Comparisons of lipids profile between *ApoE ε4* and non-*ε4* genotype in MMD patients.

A-E. MMD patients with *ApoE ε4* genotype exhibited significantly increased levels of CHO, LDL, APOB, oxLDL, and sdLDL, compared with non-*ε4* genotype patients.

*P < 0.05; **P < 0.01.

CHO, cholesterol; LDL, low-density lipoprotein; APOB, apolipoprotein B; oxLDL, oxidized low-density lipoprotein; sdLDL, small dense low-density lipoprotein.

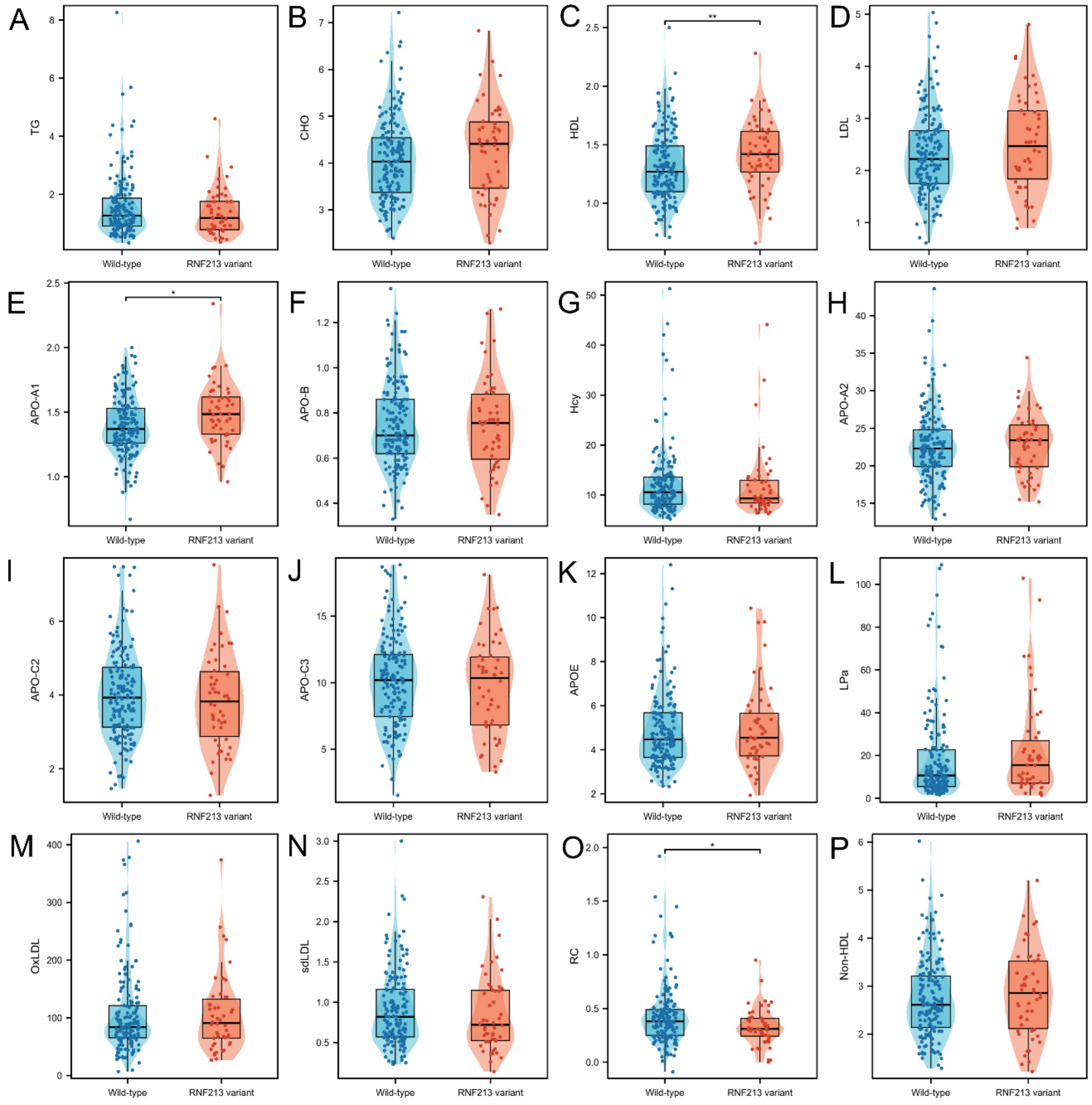

Figure S3. Comparisons of lipids profile between MMD patients with *RNF213*p.R4810K variant and wild-type genotypes.

A-P. Comparisons of TG, CHO, HDL, LDL, APOA1, APOB, Hcy, APOA2, APOC2, APOC3, APOE, LPa, oxLDL, sdLDL, RC, and non-HDL between wild-type and *RNF213* variant patients.

*P < 0.05; **P < 0.01.

TG, triglyceride; CHO, cholesterol; HDL, high-density lipoprotein; LDL, low-density lipoprotein; APOA1, apolipoprotein A1; APOB, apolipoprotein B; Hcy, homocysteine; APOA2, apolipoprotein A2; APOC2, apolipoprotein C2; APOC3, apolipoprotein C3; APOE, apolipoprotein E; LPa, lipoprotein(a); oxLDL, oxidized low-density lipoprotein; sdLDL, small dense low-density lipoprotein; RC, remnant

cholesterol; non-HDL, none high-density lipoprotein.

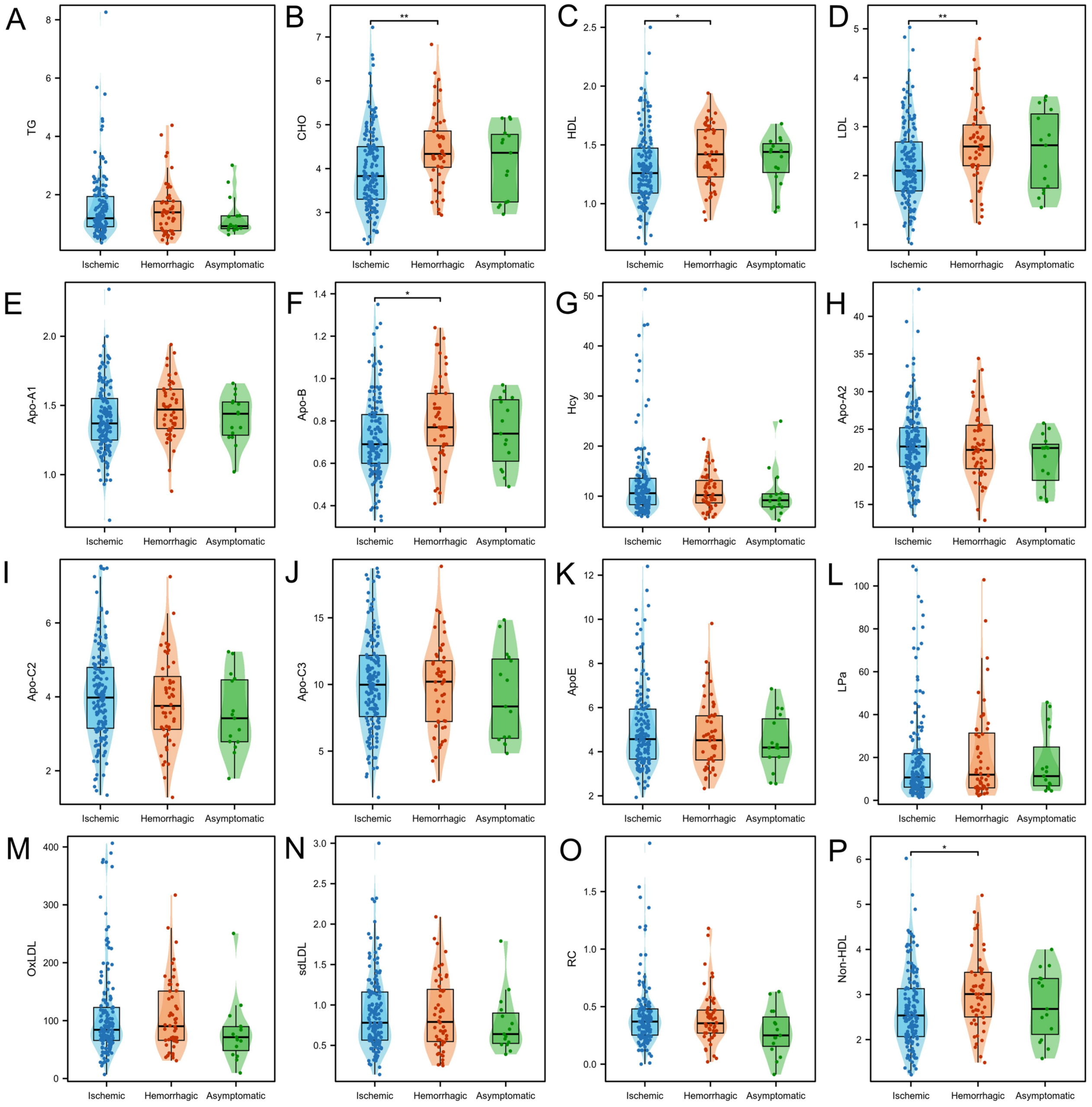

Figure S4. Comparisons of lipids profile between groups of clinical manifestations in MMD patients.

A-P. Comparisons of TG, CHO, HDL, LDL, APOA1, APOB, Hcy, APOA2, APOC2, APOC3, APOE, LPa, oxLDL, sdLDL, RC, and non-HDL between clinical manifestations in MMD patients.

$^{*}P < 0.05$; $^{**}P < 0.01$.

TG, triglyceride; CHO, cholesterol; HDL, high-density lipoprotein; LDL, low-density lipoprotein; APOA1, apolipoprotein A1; APOB, apolipoprotein B; Hcy, homocysteine; APOA2, apolipoprotein A2; APOC2, apolipoprotein C2; APOC3, apolipoprotein C3; APOE, apolipoprotein E; LPa, lipoprotein(a);

oxLDL, oxidized low-density lipoprotein; sdLDL, small dense low-density lipoprotein; RC, remnant cholesterol; non-HDL, none high-density lipoprotein.

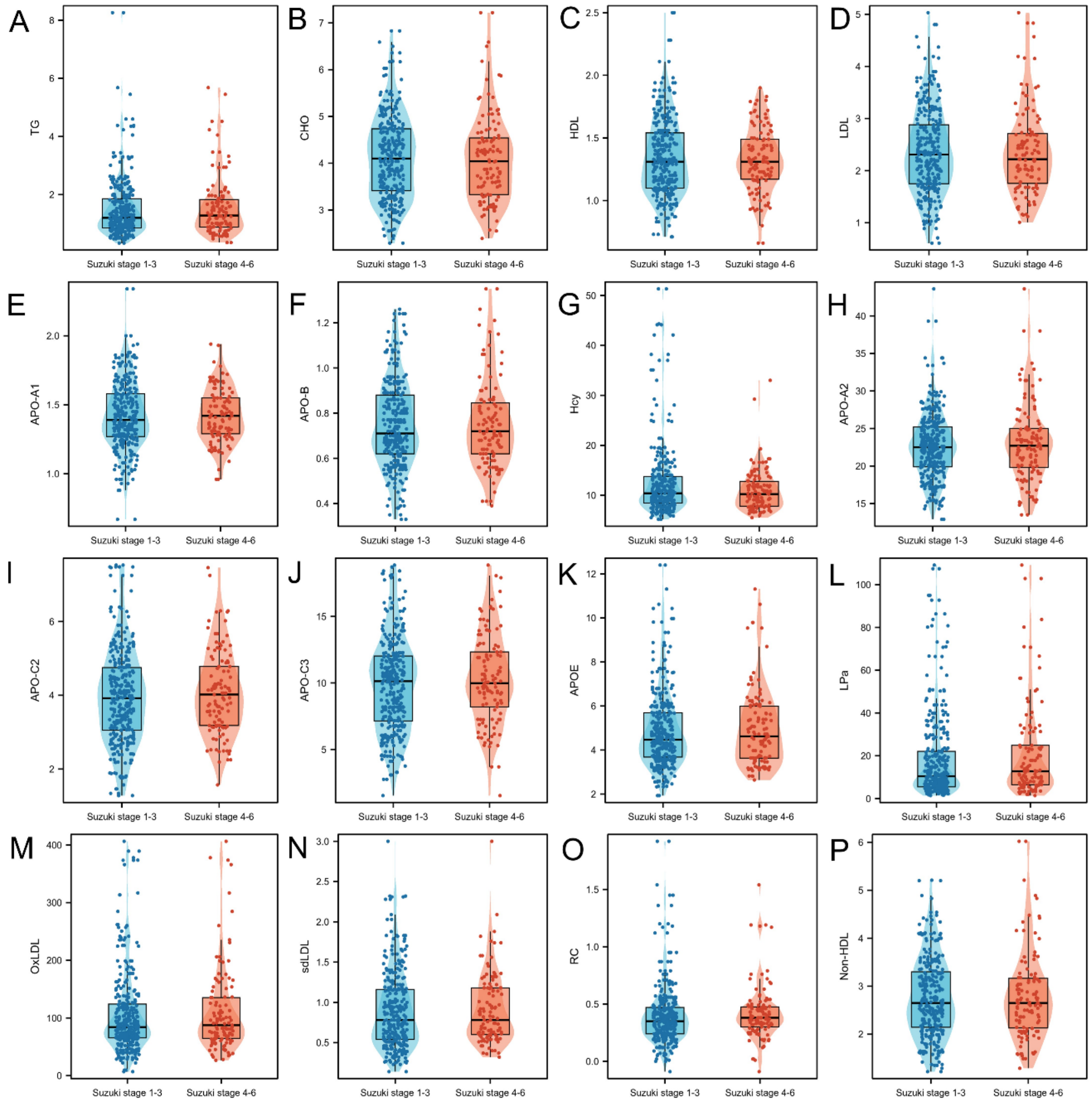

Figure S5. Comparisons of lipids profile between groups of Suzuki stage 1-3 and 4-6 in MMD patients.

A-P. Comparisons of TG, CHO, HDL, LDL, APOA1, APOB, Hcy, APOA2, APOC2, APOC3, APOE, LPa, oxLDL, sdLDL, RC, and non-HDL between Suzuki stage 1-3 and 4-6 in MMD patients.

TG, triglyceride; CHO, cholesterol; HDL, high-density lipoprotein; LDL, low-density lipoprotein; APOA1, apolipoprotein A1; APOB, apolipoprotein B; Hcy, homocysteine; APOA2, apolipoprotein A2; APOC2, apolipoprotein C2; APOC3, apolipoprotein C3; APOE, apolipoprotein E; LPa, lipoprotein(a); oxLDL, oxidized low-density lipoprotein; sdLDL, small dense low-density lipoprotein; RC, remnant cholesterol; non-HDL, none high-density lipoprotein.
